# REPRODUCTIVE HEALTH IN TRANS AND GENDER-DIVERSE PATIENTS: Gonadal tissue cryopreservation in transgender and gender-diverse people

**DOI:** 10.1530/REP-24-0253

**Published:** 2024-11-25

**Authors:** Chatchanan Ausavarungnirun, Kyle E Orwig

**Affiliations:** 1 University of Chicago/NorthShore (Endeavor Health) Pathology Residency Program, Department of Pathology and Laboratory Medicine, Evanston Hospital, Evanston, Illinois, USA; 2 Department of Obstetrics, Gynecology and Reproductive Sciences, Magee-Womens Research Institute, University of Pittsburgh School of Medicine, Pittsburgh, Pennsylvania, USA

## Abstract

**In brief:**

Gender-affirming treatments for gender dysphoria can impact fertility. This review describes the impact of gender-affirming treatments on fertility and options to preserve fertility in transgender or gender-diverse children, adolescents, and young adults.

**Abstract:**

Transgender individuals who pursue alignment with their gender identity through medical treatments or surgery face challenges to family building because the medical community lacks the understanding or infrastructure to serve the reproductive needs of transgender or non-binary people. Fertility preservation (FP) offers a crucial opportunity for the transgender community, enabling individuals to exercise autonomy over their reproductive choices. While fertility preservation has been extensively studied in other populations such as cancer patients, the unique biology and clinical care of transgender and gender-diverse (TGD) individuals have challenged the direct translation of what can be offered for cisgender individuals. Additionally, the FP services in transgender communities are reportedly under-utilized, despite the prevalent desire of TGD individuals to have children. This review aims to provide up-to-date information on the current standard of care and experimental FP options available to TGD individuals and their potential reproductive outcomes. We will also discuss the barriers to the success of FP utilization from both the biology/medical aspect and the perspectives of the TGD population. By recognizing the unique family-building challenges faced by TGD people and potential areas of improvement, appropriate adjustments can be made to better support fertility preservation in the TGD community.

## Introduction

For transgender communities, understanding the terminology is crucial for providing effective care. According to the World Professional Association for Transgender Health (WPATH) Standard of Care version 8 (SOC8) (Coleman *et al.* 2022), the term transgender or gender-diverse (TGD) is used to describe individuals whose gender identities or expressions differ from the gender typically associated with the sex assigned to them at birth. Gender identity refers to an individual’s internal sense of their gender, which is distinct from sexual orientation—defined as a person’s patterns of emotional, romantic, and sexual attraction. Gender affirmation involves recognizing and validating TGD individuals in their gender identity across social, medical, legal, and behavioral domains, or a combination of these (Poteat *et al.* 2023). Gender affirming medical and/or surgical therapy (GAMST) is the medical and surgical intervention to align a person’s body with their gender identity (Coleman *et al.* 2022). GAMST may include hormonal (gender-affirming hormone therapy: GAHT) and/or gender-affirming surgery (GAS), the latter of which may include but is not limited to genital reconstruction, removal of gonads, and surgery to enhance the secondary sex characteristics that affirm gender identity (Coleman *et al.* 2022). The evolution of terminology and diagnostic criteria shows the efforts that have been made to remove stigma from transgender communities.

Transgender individuals represent a small yet growing segment of the global population, constituting approximately 0.6% of adults and 2.7% of children and adolescents (Scheim *et al.* 2024). The reported prevalence varies depending on regions, survey methodologies, and definitions used (Reisner *et al.* 2016). More inclusive definitions of transgender, counting non-binary, gender -diverse, and gender non-conforming persons, indicate that up to 4.5% of adults and 8.4% of children and adolescents fall within this category (Scheim *et al.* 2024). In the United States, according to The Williams Institute’s 2022 report, 0.5% of adults (approximately 1.3 million individuals) and 1.4% of youth aged 13–17 (around 300,000 individuals) identify as transgender. Of the 1.3 million adults identifying as transgender, 38.5% (515,200) are transgender women, 35.9% (480,000) are transgender men, and 25.6% (341,800) are gender non-conforming (Herman *et al.* 2022). Notably, reported numbers are often higher among younger populations and may continue to rise (Zucker 2017).

TGD people show improvement in quality of life, well-being, satisfaction in one’s body image, and sexual life after receiving gender-affirming treatments (Coleman *et al.* 2022). The current recommendations for GAMST by the Endocrine Society and WPATH SOC8 can be categorized into guidelines for TGD adults/adolescents with testes or ovaries (Hembree *et al.* 2017, Coleman *et al.* 2022). GAHT for adult TGD people with testes requires both anti-androgen medications, such as Cyproterone or Spironolactone, and estrogen supplements, preferably estradiol. The protocol for adult TGD people with ovaries is testosterone monotherapy. The details of dosing and regimens vary among countries, possibly due to the availability, cost, and familiarity of clinicians with drug choices (Tangpricha & Den Heijer 2017). In adolescents, the treatment usually begins by delaying puberty with GnRH agonists (GnRHa) to allow more time for the youth to explore their gender identity and ease the distress of entering puberty before GAHT is initiated. GAHT can also later encompass puberty-blocking treatment. The recommended age to initiate GAHT, using the age of majority as previously mentioned in SOC7 – at least 16 years for GAHT and 18 years for surgery – has been updated. In SOC8, to initiate GnRHa or GAHT in the youth, they must exhibit an early sign of entering puberty (Tanner stage 2). Another important consideration is that TGD individuals must be on stable GAHT treatment for at least 6 months before GAS in adults and 12 months in adolescents unless GAHT is not desired or contraindicated. Nahata *et al*. reported the median age at which puberty blockers and cross-sex hormone therapy were prescribed was 15.0 (range: 9–18 years) and 16.0 (range: 14–18 years), respectively. The median age at the first Endocrinology visit was 15.2 years (range: 9–18 years) (Nahata *et al.* 2017).

The common indications to initiate treatment across all groups (transgender adults and adolescents of both genders) include i) having marked and sustained gender incongruence, ii) having the ability to consent, iii) that the other possible causes of gender incongruence have been ruled out, and iv) that TGD individuals fully understand the effects and consequences of treatment and thus, the benefits and risks of GAHT should be discussed, including the risk of infertility.

This review is a narrative review intended to provide up-to-date and comprehensive information regarding fertility preservation (FP) options for TGD people. We will review standard of care and experimental options for FP; implications of gender-affirming treatments for FP, as well as future reproductive options. A literature search was conducted separately for each topic using the Pubmed/MEDLINE combined database and hand search from the review references.

### Effects of GAHT on fertility

GAHT showed unpredictable and negative effects on fertility. Therefore, the Endocrine Society, WPATH, American Society for Reproductive Medicine (ASRM), and European Society of Human Reproduction and Embryology (ESHRE) recommended counseling on the impact of GAMST on fertility and options for fertility preservation prior to and periodically during GAMST (Hembree *et al.* 2017, Anderson *et al.* 2020, Ethics Committee of the American Society for Reproductive Medicine 2021, Coleman *et al.* 2022). The GAHT-prior counseling should include informing and discussing the positive and negative effects of GAHT in every aspect, not limited to reproductive health. In this section, we will discuss the effect GAHT has directly on gametogenesis and fertility.

### Effect of GAHT on spermatogenesis

GAHT effects on TGD individuals with testes are pervasive (Andrews *et al.* 2021). The severity of spermatogenesis defects can be represented using testis histopathology classification (McLachlan *et al.* 2007) and semen analysis. Histopathology findings of GAHT-exposed testicular tissues with regard to the degree of spermatogenesis are summarized in Table 1. It is worth noting that androgen cessation is usually recommended before GAS-orchiectomy with a 2–6 weeks duration depending on the center. These periods of androgen cessation may or may not have a positive impact on spermatogenesis in the testicular tissue. However, the data are inconclusive, and the duration of hormonal cessation is unknown.

**Table 1:** Effect of GAHT on spermatogenesis in TGD adults with testes prior to gender affirming surgery.

Study	Adults/ testes examined, *n*	Age* (years)	Normal *n* (%)	Hypo *n* (%)	Maturation arrest *n* (%)	Presence of germ cells *n* (%)	GAHT regimen	Duration on GAHT* (months)	Duration of cessation before GAS
Dabel *et al.* (2023)	25	28.1 (16–40)	0	0	SG:17 (68.0); SC: 5 (20.0); RS: 3 (12.0)	25 (100)§	Cyproterone acetate + estrogens	27.6 (11–66)	0–6 weeks
De Nie *et al.* (2022)	19	19.0 ± 1.5 (TS:2–3)	0	0	19 (100)	19 (100)	Triptorelin or cyproterone acetate + estrogens (may include GnRHa in adolescent group, detail not specified)	5.9 ± 1.4	4 weeks
	10	19.6 ± 1.9 (TS:2–3)	0	0	10 (100)	10 (100)		6.8 ± 1.3	0
	35	19.7 ± 1.2 (TS:4–5)	0	2	33 (94.3)	35 (100)		4.1 ± 1.8	4 weeks
	14	19.3 ± 0.7 (TS:4–5)	0	3	11 (78.6)	14 (100)		2.8 ± 0.6	0
	62	34.5 ± 12.3	0	5	52 (83.9)	57 (91.9)		2.8 ± 1.9	4 weeks
	74	36.2 ± 12.2	0	1	63 (85.1)	64 (86.5)		2.3 ± 1.2	0
Sinha *et al.* (2021)	85	39 ± 16	7 (8.2)	17 (20.0)	24 (28.2)	24 (28.2)	Mixed regimen	48 (24–60)†	NS, likely continuous
Vereecke *et al.* (2021)	97	31.19 (23.25–45.78)†	0 (acrosin-negative)	0 (acrosin-negative)	SG: 85 (87.6)‡	85 (87.6)	Cyproterone acetate + estrogen	21.7 (15.2–28.4)†	2 weeks
Jiang *et al.* (2019)	141 testes	39 (30–53)†	0	57 (40.4)	Unspecified spermatid present	114 (81)	Spironolactone, estrogen, progesterone	39 (24–65)†	2 weeks cessation of estrogen in vaginoplasty cases; the rest with continuous spironolactone or progesterone.
Jindarak *et al.* (2018)	173	26.09 ± 5.37	19 (11)	45 (26.0)	63 (36.4)	127 (73.4)	Mixed regimen	102.2 ± 55.2	4 weeks
Kent *et al.* (2018)	135	30 (18–76)†	6 (4)	0	17 (5.2)	28 (21%)	Spironolactone + estradiol and/or finasteride, progesterone	60 (12–684)†	NS
Matoso *et al.* (2018)	99 testes	33 (21–63)	0	0	SG:79 (80); SC:20 (20)	99 (100%)	Estradiol and/or spironolactone, finasteride, progesterone	6–240	NS
Schneider *et al.* (2015)	108	42 ± 12.1	26 (24.1)⁋		SG: 38 (35.19); SC:26 (24.07)	90 (83.3)	Mixed regimen	NS	Combined cohorts
	22		10 (45.5)						6 weeks
	51		22 (43.1)						2 weeks
	35		14 (40.0)						0 week

*Mean unless stated otherwise; †values are median (IQR); ⁋complete spermatogenesis; ‡Spermatogonia (MAGEA4+) positive (among these: 22 contained spermatocytes (BOLL+) and 14 contained spermatids (CREM+); §lower SG count/mm^2^ seminiferous tubule compared to cisgender age-matched control.

BOLL, boule homologue RNA-binding protein (marker for secondary spermatocytes and round spermatids); CREM, cAMP-responsive element modulator (marker for round spermatids) and acrosin (marker for acrosome visualization); GAHT, Gender-Affirming Hormone Therapy; MAGE-A4, marker for spermatogonia and early spermatocytes); NS, not specified; TGD, Transgender and gender diverse; RS, round spermatids; SC, spermatocytes; SG, spermatogonia; TS, Tanner stage.

Testicular histology findings in TGD people with testes receiving GAHT showed evidence of complete spermatogenesis (normal/hypospermatogenesis) in 0–37% of the specimens, with 21–100% presence of germ cells. Studies found no correlations between evidence of spermatogenesis and the hormonal regimen, dosage, duration on GAHT, or time off GAHT before GAS, which may be attributed to small sample sizes. Nevertheless, these findings indicate the possibility of utilizing discarded testes at the time of GAS for fertility preservation. Utilization of tissues may include but is not limited to large-volume testicular sperm extraction (TESE) on discarded testes (Niederberger 2020), and testicular tissue cryopreservation (TTC) for utilization of experimental approaches when technologies mature (please see section: Fertility preservation options – TGD people with testes – Experimental).

### Effect of GAHT on oogenesis

Regarding the ovarian histologic findings in testosterone-exposed TGD people with ovaries, some studies that reported histological findings resembling those of polycystic ovarian syndrome (PCOS) (Spinder *et al*. 1989, Pache *et al*. 1991, Grynberg *et* *al*. 2010), the disease which also involves high testosterone exposure, while other studies that found no differences in the number of primordial, early, or antral follicles compared to controls (Ikeda *et al.* 2013, De Roo *et al.* 2017, Bailie *et al.* 2023). Table 2 summarizes the important study designs from each report.

**Table 2:** Effects of GAHT on the ovarian tissues of TGD with ovaries.

Effect/study	Donors, *n*	Age (years)	Testosterone exposure duration	Control	Summary of findings
Consistent with ovarian syndrome-like change					
Pache *et al.* (1991)	17	25 (18–35)	21 months average	13 (Age: 29 (27–39))	Cortex and stromal thickening compared to control More antral follicles compared to control Multiple cystic atretic follicles
Grynberg *et al.* (2010)	112	28.9 ± 0.9	2–9 years (3.7 ± 0.6)	None	Cortex and stromal thickening More than 12 antral follicles/ovaries (PCOS features) in 89 (79.5%)
Spinder *et al.* (1989)	26	26 ± 6	9–36 months	9 age-matched patients	PCOS features in 18 (69.2%). (3/4 of stromal hyperplasia, multiple cystic follicles, collagenization of the tunica albuginea in 25 subjects (96.2%), and luteinization of stromal cells)
Comparable oocyte distribution to control, no PCOS features					
Ikeda *et al.* (2013)	11	27–38	17 months–14 years (median: 38 months)	10 age and BMI-matched oncology patients	No differences in oocyte distribution numbers compared to control Cortical and medullary hyperplasia noted
De Roo *et al.* (2017)	40	24.30 ± 6.15	14.5 ± 6.6 months	Compare with previously published normal values No internal control group	No differences in oocyte distribution numbers compared to control Cortical and medullary hyperplasia noted
Bailie *et al.* (2023)	8	27.6 ± 1.7	18 months–10 years	31.8 ± 1.5 healthy donors	Higher proportion of non-growing ovarian follicles, higher levels of DNA damage. More growing follicles in transgender ovaries compared to control, but follicle health further deteriorated

BMI, body mass index; PCOS, Polycystic Ovarian Syndrome; GAHT, Gender-Affirming Hormone Therapy; SD = standard deviation; TGD = Transgender and gender diverse.

### Fertility preservation options

There are still no standard guidelines regarding FP choices for TGD individuals. This may be due to limited evidence to make the recommendations. We will review standard-of-care fertility preservation options that have been offered to TGD individuals and experimental options that are offered at very few centers with Institutional Review Board (IRB) approval. It is very important to note that, unlike in cancer patients, FP interventions are not usually offered until Tanner stage 2 (approximately 11 years old in females and 11.5 years old in males) is reached as this stage of development is required for GnRHa/GAHT initiation. Therefore, we will focus our review on findings from the peripubertal period and older.

### Fertility preservation options for TGD people with testes

Two fertility preservation options are possible for TGD people with testes. The established and standard of care option is to cryopreserve a semen sample with sperm. Cryopreserved sperm can be thawed in the future to fertilize partner or donor eggs and establish a pregnancy. This method has extensive evidence supporting its use in adult cisgender males and is the only recommended standard protocol for adults facing gonadal threats, such as chemotherapy or total body radiation (Gassei *et al.* 2017, Martinez 2017, Oktay *et al.* 2018, Practice Committee of the American Society for Reproductive Medicine 2019). The second option is TTC, which is typically reserved for prepubertal patients who are not producing sperm. TTC is experimental both for cisgender patients with a cancer diagnosis or TGD patients with a gender dysphoria diagnosis because there is no evidence yet that those tissues can be matured in the future to produce sperm. While many centers around the world provide TTC to cancer or bone marrow transplantation patients who are at risk of infertility, very few provide this service to TGD individuals who cannot or will not interrupt GAHT to collect and freeze a semen sample with sperm. TESE can be offered to TGD people with testes who are going to gender-affirming surgery, as the testes are typically removed during the GAS process and would otherwise be discarded. However, the long-term impact of GAHT prior to GAS is not known. Table 3 summarizes fertility preservation outcomes by semen collection and TESE based on age groups and history of GnRHa/GAHT exposure.

**Table 3:** Fertility preservation (semen analysis or TESE outcomes) in TGD people with testes.

**Age group**	**GAHT exposure status**	**Technique used**	**Results**	**References**
Adult	No prior GnRHa and GAHT exposure	Semen collection	Poor semen parameters compared to referenced cisgender samples	Adeleye *et al*. (2019*b*); Rodriguez-Wallberg *et al*. (2021*a*); Hamada *et al*. (2015); Barda *et al*. (2023); de Nie *et al*. (2020); Li *et al.* (2018)
			Poor semen parameter in post-thawed samples	de Nie *et al*. (2020); Hamada *et al.* (2015)
	With continued GnRHa/GAHT at collection	Semen collection	Low semen parameters compared to previously-used GAHT and GAHT-naïve	Adeleye *et al*. (2019*b*)
	Stop GnRHa/GAHT at collection	Semen collection	Semen parameters poorer than GAHT-naïve TGD samples	Rodriguez-Wallberg *et al*. (2021*a*)
			Semen parameters comparable with GAHT-naïve TGNB samples.	Adeleye *et al*. (2019*b*); Barda *et al*. (2023)
			Semen parameters higher than continuously-used GAHT	Adeleye *et al*. (2019*b*)
		Semen collection or testicular sperm extraction	Natural conceptions reported in 3/9 cases; Viable sperm retrieved from all 9 cases by semen collection or testicular sperm extraction.	de Nie *et al.* (2023)
Peripubertal	No prior GnRHa and GAHT exposure	Semen collection (16-24-year-old TGDs)	Normal semen parameters except for low percentage (3%) of normal morphology compared to normal reference per Modified Kruger criteria (>13%) in group with mean age 19.5	Barnard* et al*. (2019)
		Testicular sperm extraction (13-17-year-old TGDs)	Successful sperm retrieval (68%, 17/25)	Peri *et al*. (2021)
	With continued GnRHa/GAHT at collection	No data	No data	No data
	Stop GnRHa/GAHT at collection	Semen collection (age 17.5 at GnRHa initiation, age 18 at retrieval, n=1)	12 sperm (2 motile) found 3 months after suspending GnRHa; Normal semen sample 5 months after suspending GnRHa	Barnard *et al.* (2019)
		Semen collection (age 18 at initiation, age 19 at retrieval, n=1)	Azoospermic at 4 months after suspending GAHT	Barnard *et al.* (2019)

GnRHa, Gonadotropin releasing hormone agonist; GAHT, gender-affirming hormone therapy

## Standard of care FP options for TGD people with testes

### Adult

#### Before the initiation of GnRHa or GAHT

Although sperm cryopreservation is recommended in adults who can produce sperm, the collection of semen via masturbation may cause psychological distress and exacerbate gender dysphoria in some cases (Reckhow *et al.* 2023). Also, there is a high prevalence (47%) of orgasmic dysfunction in TGD people with testes, even before GAHT (Kerckhof *et al.* 2019). In such cases, alternative ways to obtain sperm, such as Electro- or vibratory stimulation, TESE, Testicular Sperm Aspiration (TESA), or Epididymal Sperm Aspiration (PESA), among others, may be offered (Esteves *et al.* 2011). Adult TGD people with testes also had poorer semen parameters (sperm concentration, total motile sperm count, and/or morphology) compared to the WHO-referenced male or healthy cisgender male control group even before GAHT (Li *et al.* 2018, De Nie *et al.* 2020, Rodriguez-Wallberg *et al.* 2021a) (Table 3). Although not directly evaluated in these reports, poor sperm parameters before GAHT were thought to be attributed to lifestyle or environmental factors such as the tucking of the testicles (Trussler and Carrasquillo 2020). Additionally, cryopreserved semen from TGD individuals before GAHT showed that only 26% of the post-thawed samples were of adequate quality for intrauterine insemination (IUI), the cheapest and simplest assisted reproductive technology (ART) (De Nie *et al.* 2020, Hamada *et al.* 2015). Therefore, even when pursuing FP before GAHT, TGD patients with testes may need to plan for more expensive ARTs in the future, such as *in vitro* fertilization (IVF) with intracytoplasmic sperm injection (ICSI). However, Hamada and colleagues did report a case of fertilization and pregnancy using a single transwoman’s cryopreserved sperm for IUI in a surrogate mother (Hamada *et al.* 2015).

#### After the initiation of GnRHa or GAHT

TGD people with testes whose GAHT treatment has been initiated without prior fertility preservation can collect sperm via the same means as the GAHT-naive group, opening up more flexibility to those who were undecided, prioritized initiation of GAHT, or simply changed their plan on family building. There is histologic evidence of complete spermatogenesis (Table 1) and evidence to suggest that sperm can be recovered in the semen or by TESE after temporary cessation of gender-affirming treatments in some cases (Table 3). Therefore, the state of GnRHa or GAHT should not preclude fertility preservation.

#### Adolescent

Recommendations for FP choice in adolescent TGD people with testes still sperm cryopreservation. However, this may not be feasible in adolescents under 15 years old due to the high prevalence of azoospermia (no sperm in the ejaculate). A recent study in peripubertal cancer patients reported azoospermia in 66.7% of 12-year-olds, 31.3% of 13-year-olds, and approximately 10% of 14–17-year-olds, decreasing to 0% in 18–19-year-olds (Halpern *et al.* 2019). Even if no sperm are found in the ejaculate, it is sometimes possible to retrieve sperm directly from the testis by TESE. Peri and colleagues reported that sperm recovery via TESE was successful in 68% of patients in the 13–17 year-old range with no prior gender-affirming treatments (Peri *et al.* 2021) (Table 3).

### Experimental: TTC

TTC has been offered and studied as an experimental FP approach in prepubertal cancer patients worldwide with the expectation that these tissues can be matured in the future to produce sperm from resident spermatogonial stem cells (SSCs) (Tran *et al.* 2022). Our center has extended this experimental FP option to young TGD patients (NCT05829928). This protocol is separate from our cancer patient TTC protocol because the risks and benefits for TGD patients are different than those for cancer patients. Our center is approved to cryopreserve testicular tissues for patients who have a diagnosis of gender dysphoria and are referred by their physician for fertility preservation. Patients must be ≥9 years old, getting ready to start or already on gender-affirming treatments, and unwilling or unable to delay or interrupt GnRHa or GAHT to collect sperm. If patients are 12 years or older, we provide the option to search a portion of the tissue for sperm, similar to TESE. However, the majority of the tissue is cryopreserved with the expectation that SSCs in the tissue have the potential to produce sperm in the future. Peri and colleagues reported retrieval of sperm from the testicular tissues of young TGD patients who were Tanner stage 3 or higher and when testis volumes were greater than 10–12 mL. Age, hormone levels, and previous gender-affirming treatments were not reliable determinants of whether sperm could be retrieved from testicular tissues (Peri *et al.* 2021). Therefore, Tanner staging and testis volume data may be useful in counseling young TGD patients about the potential future uses of their cryopreserved testicular tissue. Several studies showed the presence of undifferentiated germ cells (stem and progenitor spermatogonia) in TGD testicular tissue regardless of GAHT history, showing the potential utility of cryopreserved testicular tissues in this group (Table 1). This may suggest that suspension of gender-affirming treatments is not necessary prior to cryopreservation of testicular tissue with SSCs. TTC may also be possible when testes are being removed for GAS. However, there is no data on the function of germ cells that may remain in that tissue after long-term GAHT treatment. Studies in animal models have shown different ways to utilize the cryopreserved testicular tissue in both tissue-based and cell-based approaches (reviewed in (Tran *et al.* 2022)). Future utilization of tissues requires different considerations than in cisgender cancer survivors because TGD people may not want the tissue or cells transplanted back into their bodies or want to go through puberty in the gender that would be required to mature their tissues/cells inside their bodies. Methods to mature testicular tissue or cells outside the body to produce sperm (see below) may be required but are in very early stages of development.

## Potential uses of cryopreserved testicular tissues in reproduction: considerations for TGD individuals

### Testicular tissue or cell transplantation

Brinster and colleagues pioneered the method of spermatogonial stem cell transplantation more than three decades ago. Testicular cells (including SSCs) were injected into the seminiferous tubules of the testes where they regenerated spermatogenesis with sperm that were competent to fertilize and produce offspring (Brinster and Zimmermann 1994, Brinster and Avarbock 1994). Donor SSCs of any age are competent to regenerate spermatogenesis. In addition, cells that were thawed after 14 years of cryostorage could regenerate spermatogenesis (Wu *et al.* 2012), which is relevant in the context of fertility preservation in young patients. Testicular tissue grafting is an alternative approach that involves transplanting intact pieces of testicular tissue under the skin. Fresh or cryopreserved immature testicular tissue can be matured over several months *in vivo* and then recovered and dissected to release sperm that are competent to fertilize by IVF with ICSI and produce offspring (Honaramooz *et al.* 2002, Schlatt *et al.* 2003, Shinohara *et al.* 2002, Fayomi *et al.* 2019). Testicular tissue grafting is usually performed in castrated recipients, which may be germane to TGD patients after GAS. This approach works only with immature (prepubertal) testicular tissues and not adult tissues (Arregui and Dobrinski 2014). It is not known whether testicular tissues from TGD patients where spermatogenesis is suppressed by gender-affirming treatments would function more like adult tissues or immature prepubertal tissues in this context. However, it is noteworthy that when spermatogenesis was suppressed in mice with acyline (GnRH antagonist) prior to transplantation, grafts survived and produced spermatogenesis (Arregui *et al.* 2012).

Spermatogonial stem cell transplantation and testicular tissue grafting are mature technologies that have been replicated in numerous animal models, including nonhuman primates (reviewed in (Tran *et al.* 2022)) and may be ready for translation to the human clinic. However, as indicated above, TGD patients may not want their testicular tissues or cells transplanted back into their body or to go through male puberty with testosterone production, which is necessary for spermatogenesis to occur from transplanted testicular cells or tissues. Below, we review *ex vivo* approaches to mature testicular tissues or cells and produce sperm. These methods are at a much earlier stage than the transplant approaches described above but may have valuable applications for TGD patients who have cryopreserved their testicular tissues.

### Xenotransplant into SCID/Nude mice or other animal hosts

An alternative to autologous transplantation is testicular tissue grafting into an animal host. Testicular tissue from several species (reviewed in (Tran *et al.* 2022)) can be transplanted under the dorsal skin or scrotal skin of immune-deficient SCID or nude mice and matured to produce sperm as well as offspring in rabbits (Shinohara *et al.* 2002), pigs (Nakai *et al.* 2010) and monkey (Liu *et al.* 2016). In humans, the most advanced germ cells produced by this technique were premeiotic spermatocytes, which have been reported for both immature and adult as well as fresh or frozen human testicular grafts (References can be reviewed in Table 4). It is unclear why prepubertal monkey testicular tissues can be matured to produce sperm in a mouse host, while human testicular tissues cannot. Perhaps other animal hosts, such as immune-deficient pigs (Boettcher *et al.* 2018) will support better development of human tissues. The risk of transmitting viruses or other xenobiotics from the animal host to the patient must be carefully considered (Kimsa *et al.* 2014, 2017). However, it is noteworthy that pigs are actively being developed as organ donors for human patients (Kozlov 2024).

**Table 4:** Developing technologies for maturing patient testicular tissues/cells and producing sperm outside the patient’s body. Evidence in human studies.

**Tissue source**	**Technique**	**Methods**	**Results**	**Reference**
Cisgender prepubertal tissue	Tissue- based	Xenotransplant into SCID or nude mice		
Fresh tissue into dorsal skin				
From 10-11-year-old donors, *n*=3			Spermatogonia	Goossens *et al.* (2008)
From 3-9-year-old donors, *n*=3			BOLL+ spermatocytes	Ntemou *et al.* (2019)
Fresh tissue into scrotum				
From 5-year-old donor, *n*=1			Spermatogonia	Van Saen *et al*. (2011)
From 12-13-year-old donors, *n*=2			Spermatocyte	Van Saen *et al.* (2011)
From 2-12-year-old donors, *n*=10			Spermatocyte	Poels *et al.* (2013)
From 3-9 -ear-old donors, *n*=3			Spermatocyte	Ntemou *et al.* (2019)
Frozen prepubertal tissue into scrotum				
From 3-13-year-old donors, *n*=3			Spermatogonia	Van Saen *et al*. (2011)
From 2-12-year-old donors,* n*=11			Spermatogonia	Wyns *et al*. (2007)
From 2-15-year-old donors,* n*=6			Spermatogonia	Poels *et al*. (2014)
From 7-14-year-old donors, *n*=5			Spermatocyte	Wyns *et al*. (2008)
From 2-12-year-old donors, *n*=10			Spermatocyte	Poels *et al*. (2013)
Cisgender adult tissue				
Fresh tissue into dorsal skin			Degenerated tissue	Schlatt *et al*. (2006)
			Spermatogonia	Geens *et al*. (2006)
Fresh adult tissue into scrotum			Spermatocyte	Van Saen *et al*. (2011)
Frozen adult tissue into scrotum			Spermatocyte	Van Saen *et al*. (2011)
Cisgender immature tissues (age 6-14)	Tissue- based	IVM with testicular tissue culture		
Used fresh			Spermatogonia	Portela *et al*. (2019)
Used frozen			Spermatogonia	Portela *et al.* (2019), de Michele *et al*. (2017)
			SYCP3+ primary spermatocytes	Medrano *et al*. (2018), Younis *et al*. (2023)
			Round spermatid	de Michele *et al*. (2018)
Cisgender adult tissue				
Used Fresh			Spermatogonia	Jorgensen *et al.* (2014)
Transgender adult tissue				
Fresh and cryopreserved adult GAHT-exposed testicular tissue			No progression of spermatogenesis after 2 weeks in culture	Komeya *et al.* (2021)
Cisgender prepubertal and adult cells	Cell-based	*De novo* testicular morphogenesis (organoid culture)		
Fresh pubertal (age 15) and adult testicular cells	SC- based, or SC-free transwell		Mitotically-active germ cells, normal somatic cells function and arrangement	Baert *et al.* (2017)
Frozen prepubertal testicular cells	Matrigel		Inverted organization of spermatogonia and somatic cells	Sakib *et al*. (2019)
Fresh and frozen adult testicular cells	ECM		Spermatogonia clusters, normal somatic cells function and arrangement	Baert *et al*. (2015)
			PRM2+ elongated spermatids	Pendergraft *et al*. (2017), Nikmahzar *et al*. (2023)

TGNB, transgender and non-binary; GnRHa, gonadotropin-releasing hormone agonist; GAHT, gender-affirming hormone therapy, SYCP3 +, synaptonemal complex protein 3 (marker for primary spermatocytes); SCID, severe combined immunodeficiency;, BOLL , boule homologue RNA-binding protein (marker for secondary spermatocytes and round spermatids); ECM, extracellular matrix; IVM, *in vitro* maturation; SC, scaffold.

### *In vitro* maturation with testicular tissue organ culture

Sato and colleagues pioneered a method for culturing immature mouse testicular tissues at the air-liquid interface. Tissues matured over several weeks in culture and produced sperm that were competent to fertilize and produce offspring (Sato *et al.* 2011). Like testicular tissue grafting, this approach only works with immature testicular tissues; and it is not yet known whether it would work with testicular tissues where spermatogenesis is suppressed by gender-affirming treatments. Several groups have reported culturing human testicular tissues at the air-liquid interface. Tissues could be maintained for weeks to months with the maintenance of spermatogonia and occasional differentiation to produce spermatocytes or spermatids but not sperm (Medrano *et al.* 2018, De Michele *et al.* 2018, Portela *et al.* 2019, Younis *et al.* 2023). Komeya and colleagues reported that GAHT-exposed testicular tissues could be maintained for 2 weeks in culture, but the number of germ cells declined over that time (Komeya *et al.* 2021). Testing the fertilization potential of experimentally derived human sperm, using this approach or others, is necessary to demonstrate safety and feasibility, but raises ethical concerns and is challenged by restrictive funding or laws in some states and countries.

### *De novo* testicular morphogenesis in an animal host or organoid culture

Heterogeneous testis cell suspensions have the remarkable ability to reform seminiferous tubules, both *in vivo* and *ex vivo*. Testis cells from mice, sheep, and pigs can be pelleted and transplanted under the skin of mouse recipients, where they reform into seminiferous tubules, which sometimes contain spermatids and/or sperm (Honaramooz *et al.* 2007, Kita *et al.* 2007, Arregui *et al.* 2008). The fertilization potential of those sperm has not been tested, and to our knowledge, *in vivo* *de novo* testicular morphogenesis has not been reported with human testis cells. Many groups have described methods for *de novo* testicular morphogenesis *ex vivo*, but none have yet produced sperm or offspring. Sakib and colleagues reported a microwell aggregation approach to produce 3D testicular organoids from neonatal or prepubertal testicular cell suspensions of mice, pigs, monkeys, and humans. The tubules formed inside out and contained spermatogonia but did not support complete spermatogenesis (Sakib *et al.* 2019). Two studies reported human testicular organoids from adults (15+ years) formed in the human testicular extracellular matrix (htECM). Baert and colleagues seeded heterogeneous prepubertal or adult human testis cell suspensions onto a 3-dimensional htECM scaffold that was shaped in the form of a tubule (Baert *et al.* 2017). Pendergraft and colleagues used a hanging-drop method to induce organoid formation from cultured adult human spermatogonia mixed with immortalized human Sertoli and Leydig cells suspended in a hydrogel of htECM (Pendergraft *et al.* 2017). Both approaches led to the production of organoids including germ cells and somatic cells, but neither approach produced seminiferous tubule-like structures (Pendergraft *et al.* 2017, Baert *et al.* 2017). The Pendergraft study reported elongated spermatids, but since the starting point was adult tissues, it is impossible to determine whether those post-meiotic spermatids arose in culture or were already present in the original cell suspension (Pendergraft *et al.* 2017) (Table 4).

## Standard of care FP options for TGD people with ovaries

### Adult

#### Before the initiation of GAHT

Ovarian stimulation and oocyte cryopreservation can be done the same way as for cisgender females. Maxwell and colleagues reported four successful four live births in two couples utilizing cryopreserved oocytes from GAHT-naive adult TGD with ovaries, followed by fertilization with donor sperm and embryo transfer into cisgender, sexually intimate, female partners (Maxwell *et al.* 2017, Adeleye *et al.* 2019a). TGD people with ovaries (with and without prior testosterone exposure) produced a similar number of oocytes, with a similar maturity rate as age/BMI-matched cisgender women (Adeleye *et al.* 2019a, Leung *et al.* 2019) (Table 5).

**Table 5:** Oocyte cryopreservation fertility outcome in TGD with ovaries

**Age group**	**GAHT exposure status**	**Technique used**	**Results**	**References**
Adults	No prior GAHT exposure	Cryopreserved oocytes and/or embryos	Live births	Maxwell *et al.* (2017)
		Fresh oocytes	3 pregnancies	Adeleye *et al*. (2019*a*)
	With continued GAHT at collection	Fresh transfer with reciprocal IVF	Live birth	Greenwald *et al*. (2022)
		Fresh transfer	Live birth	White *et al*. (2024)
		Oocyte retrieval	Successful oocyte retrieval	Stark & Mok-Lin (2022); Gale *et al*. (2021); Cho *et al*. (2020)
	Stop GAHT at collection	Fresh transfer and embryo cryopreservation)	2 pregnancies	Adeleye* et al*. (2019*a*)
		IUI with donor sperm, IVF and reciprocal IVF, no freezing	5 live births	Ghofranian *et al*. (2023)
		Fresh or frozen transfer	7 live births	Leung *et al*. (2019)
Pubertal/ adolescent	No prior GAHT exposure	Oocyte retrieval	Successful oocyte retrieval	Chen *et al.* (2018); Barrett* et al*. (2022); Insogna* et al*. (2020)
	With continued GAHT at collection	No data	No data	No data
	Stop GAHT at collection	Oocyte retrieval in GnRHa-only, and who had history of prior testosterone use	Successful oocyte retrieval	Insogna *et al*. (2020)

GAHT, gender-affirming hormone therapy; IUI, intrauterine insemination

#### After the initiation of GAHT

Oocyte cryopreservation and embryo cryopreservation can be offered even after the initiation of GAHT. However, Adeleye *et al*. reported that the number of oocytes retrieved from GAHT naive TGD with ovaries was higher than in the group with prior GAHT who had suspended testosterone treatment for a median time of 6 months (Adeleye *et al.* 2019a). The main question in this scenario is whether or not to discontinue testosterone supplements before oocyte retrieval. Testosterone cessation has traditionally been encouraged to ensure a good oocyte retrieval outcome, and the duration recommended is at least 3 months or until the return of menstruation (De Roo *et al.* 2016, Armuand *et al.* 2017). However, the necessity to suspend GAHT and resume menstruation requires further investigation because GAHT interruption can cause distress in TGD people (Armuand *et al.* 2017, Greenwald *et al.* 2022) (Table 5).

Ovarian tissue cryopreservation (OTC) is no longer considered experimental by the ASRM (Practice Committee of the American Society for Reproductive Medicine 2019), based in part on the evidence of more than 130 live births from transplanted ovarian tissues (Donnez and Dolmans 2017). However, that guidance was based almost entirely on data from survivors of cancer or bone marrow transplantation who were adults at the time of OTC. Data on the transplantation potential of ovarian tissues that were cryopreserved during childhood or from TGD individuals on GAHT are limited or absent, respectively. Thus, it is reasonable to offer OTC as an experimental option until more transplantation and live birth data can be accumulated for those populations.

### Adolescent

#### Before GAHT initiation

Oocyte cryopreservation is the standard fertility preservation option for hormone-naive adolescent TGD people with ovaries. OTC could be offered at the time of GAS, but those patients have usually already initiated GAHT according to WPATH SOC 8 recommendations. According to WPATH SOC 8, GAS is usually recommended after 6 months of stable GAHT in adults and 12 months in youth unless the GAHT is not desired or contraindicated. This means that, in most cases, OTC with simultaneous GAS is generally not possible unless GAHT has begun (Amir *et al.* 2020a). Embryo cryopreservation, which requires partner sperm, is not usually offered in adolescents. OTC for fertility preservation is not generally offered as a stand-alone option to adolescent TGD patients with ovaries, although it is offered at our center as an experimental protocol (NCT05863676).

## After initiation of gender affirming treatments

Two studies have shown successful oocyte retrieval in adolescent TGD with ovaries who had GnRHa only and who had prior testosterone use (Insogna *et al.* 2020, Barrett *et al.* 2022). Considerations for embryo freezing and OTC are the same as described above. Our center does not require the cessation of GnRHa or GAHT prior to OTC. This may be a consideration for TGD people who do not want to interrupt their gender-affirming treatments for fertility preservation.

## Potential uses of cryopreserved ovarian tissues in reproduction: Considerations for TGD individuals

### Autologous transplantation

Cryopreserved ovarian tissue can be transplanted back to the donor at the ovary or pelvic site. Transplanted ovarian tissues can restore hormonal and reproductive function, including the possibility of *in vivo* conception and pregnancy. There have been more than 180 live births after transplantation of cryopreserved ovarian tissues using *in vivo* conception or IVF (Donnez and Dolmans 2017, Gellert *et al.* 2018, Practice Committee of the American Society for Reproductive Medicine 2019, Khattak *et al.* 2022) Transplantation of ovarian tissues that were cryopreserved in prepuberty, adolescence, or adulthood has resulted in live births (Table 6). To our knowledge, there are no reports of ovarian tissue transplantation in TGD individuals. While ovarian tissue transplantation is a robust technology, it probably requires GAHT cessation and the production of estrogen from developing follicles. However, we note that ovulation appears to be possible while still on testosterone treatment (Asseler *et al.* 2024, Stark and Mok-Lin 2022, Gale *et al.* 2021, White *et al.* 2024, Greenwald *et al.* 2022). Additional research may reveal protocols that enable follicle development in transplanted ovarian tissues without compromising gender-affirming medical treatments.

**Table 6:** Technology maturity of potential experimental fertility preservation approach for transgender men

**Patient population**	**Method**	**Technique**	GAHT cessation*	**Results**	**References**
Cisgender	ALT	Tissue-based	Yes		
Adult tissue				Live births	Reviewed in Gellert *et al*. (2018), Donnez & Dolmans (2017), Khattak* et al*. (2022)
Cisgender prepubertal and adolescent tissue				Live births	Demeestere *et al*. (2015), Matthews *et al*. (2018), Rodriguez-Wallberg *et al.* (2021*b*)
Transgender				No data	No data
Cisgender	OTO/IVM	Cell-based	No		
Patients with cancer or ovarian neoplasm				Live births	Segers *et al.* (2020)
				Live birth^†^	Kedem *et al*. (2018), Uzelac *et al*. (2015), Prasath *et al*. (2014)
				50-76.9% fertilization rate; Pregnancy rate not reported due to no utilization	Reviewed in Mohd Faizal *et al*. (2022)
				Successful oocyte aspiration during cesarean section	Hwang *et al*. (1997)
Benign pelvic AVM				Pregnancy	Segers *et al*. (2015)
TGD with ovaries					
Adult				Normal spindle after thawing	Lierman *et al.* (2017)
				Poor embryonic progression after fertilization	Lierman *et al*. (2021)
				Poor embryonic progression overcome by spindle transfer	Christodoulaki *et al.* (2023)

ALT, autologous transplantation; AVM, arteriovenous malformation; TGD, transgender and gender diverse individuals; OTO/IVM,ovarian tissue oocyte/in vitro maturation.

*GAHT cessation at the time of fertility restoration; †Live birth rate after embryo transfer = 43%

### Ovarian tissue oocyte followed by *in vitro* maturation (OTO/IVM)

OTC can be performed prior to the initiation of GAHT, during GAHT, or concomitantly with ovariectomy as a part of the GAS. During ovarian tissue processing, the outer cortex of the ovary, which contains primordial follicles, is dissected away from the inner medulla and then cut into strips for cryopreservation. Small antral follicles that are present in the medulla are released into the dissection media and are usually discarded. Cumulus-oocyte complexes (COCs) retrieved from these medullary antral follicles can potentially be matured to produce MII oocytes or embryos that can be cryopreserved in parallel with the ovarian tissues (Cadenas *et al.* 2023). This approach does not require stimulation with exogenous hormones because the final steps of egg maturation occur *in vitro*. The birth of five healthy infants has been reported using this approach (Prasath *et al.* 2014, Uzelac *et al.* 2015, Segers *et al.* 2020) (Table 6). While data are limited in TGD with ovaries, studies showed normal oocyte distribution across all layers of ovarian tissue (De Michele *et al.* 2017, Bailie *et al.* 2023), though one study indicated higher yH2AX staining, a marker for DNA breaks, in primordial germ cells compared to cisgender control (Bailie *et al.* 2023). The COCs that were extracted from the medulla resulted in MII oocytes after IVM with 87% normal spindle structure, also indicating the possibility of using ovarian tissue oocytes with IVM (OTO-IVM) in TGD people with ovaries and a history of GAHT. However, poor embryo development was noted in GAHT-exposed *in vitro*-matured ovarian tissue oocytes recovered at the time of GAS during ovarian tissue processing (Lierman *et al.* 2021, Christodoulaki *et al.* 2023) and may be improved by spindle transfer (Christodoulaki *et al.* 2023). Thus, OTC earlier in transition before exposure to GAHT may be beneficial.

### *In vitro* growth of primordial follicles followed by IVM in multistep culture

Cortical strips contain primordial follicles that can be extracted for *in vitro* development of primordial follicles (primordial follicle to antral follicle) and IVM (immature antral follicle to MII oocytes). The resulting MII can then be used for cryopreservation or fertilized for embryo transfer/cryopreservation. This approach has been studied as an alternative for cancer patients where the chance of reintroducing cancer is high. It shows promise in TGD with ovaries whose primordial follicles are retained in the cortical strip, and the reversal of GAHT is not required at the time of fertility restoration. While *in vitro* maturation from primordial follicles to mature MII oocytes and preimplantation embryos was described more than a decade ago in mice (Jin *et al.* 2010), IVM to mature MII oocytes has only been achieved when starting from growing primary and secondary follicles in primates and humans (Xu *et al.* 2013) (reviewed in (Hu *et al.* 2023)). An artificial ovary that reconstitutes the ovarian microenvironment *ex vivo* may provide a path forward (Amorim and Shikanov 2016, Laronda *et al.* 2017).

### Barriers to successful fertility restoration in TGD communities

**Figure 1:**
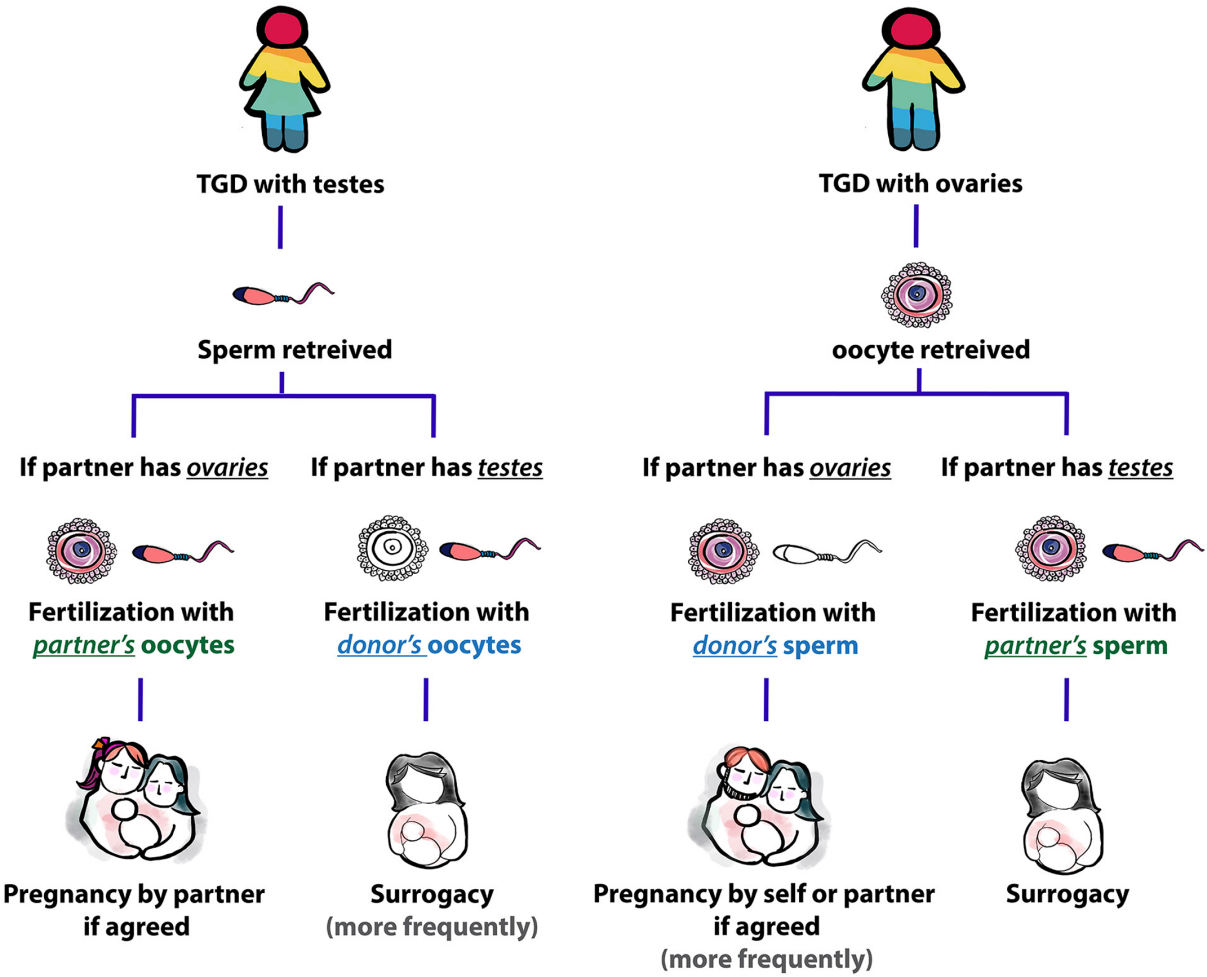
Journey of TGD people to have a biological child.

Reproductive desire and/or interest in family building is high among transgender people, both adults and youth, but FP services are reportedly under-utilized in many countries around the world. A meta-analysis using 76 studies showed 48.7–67.0% of transgender adolescents and 18.4–82.1% of transgender adults desired children, but FP utilization rates were 2–4% (Stolk *et al.* 2023). It is noteworthy that successful sperm/oocyte/gonadal tissue cryopreservation is only the beginning of the journey to successful family building. Multidisciplinary teams are required to ensure that TGD people have access to fertility preservation care and develop technologies that will enable them to use their cryopreserved cells or tissues for family building with minimal disruption to gender-affirming care. Figure 1 shows the journey of a TGD person to have a biological child. For TGD people with testes, ejaculated sperm or sperm from testicular tissues can be used to fertilize partner or donor eggs using standard Assisted Reproductive Technologies (ARTs). If the partner has testes, egg donation for same-sex couples and surrogacy is often required. For TGD people with ovaries, fertility seems to be less affected by hormonal treatment compared to TGD people with testes. Once oocytes are collected by hormonal stimulation or from ovarian tissues, partner or donor sperm and ART are required for fertilization and conception. If the partner has ovaries, sperm donation for same-sex couples will be needed. Surrogacy is possible but may not be needed if the partner is a biological female who will carry the pregnancy.

## Conclusion

The impacts of gender-affirming treatments on fertility and family building should be discussed before and throughout treatment. Explaining options for fertility preservation and restoration provides a sense of reproductive autonomy, even if the patient is unsure of their family-building goals. Like FP for cancer patients, it is important to start these discussions early while the medical and research communities are still learning the impacts of gender-affirming treatments on the ovaries, testes, eggs, and sperm. Early intervention for FP may be important in some cases. For fertility preservation to accomplish its purpose (which is to allow TGD people to have biological children if they want to), it takes multidisciplinary teams, ranging from pediatric and adult endocrinologists, mental health professionals, reproductive medicine experts and scientists. Laws that support same-sex parenting, egg/sperm donation for same-sex couples, and surrogacy will help ensure that TGD people have the same access to reproductive care as cisgender people. There is an unmet need for counseling and education to cisgender and TGD communities about the availability, accessibility, and feasibility of fertility preservation and fertility restoration options for all people as well as the specific challenges and opportunities for TGD people.

## Declaration of interest

The authors declare that there is no conflict of interest that could be perceived as prejudicing the impartiality of the study reported.

## Funding

This work was supported by anonymous donor funds to Magee-Womens Research Institutehttp://dx.doi.org/10.13039/100012386 and Foundation.

## Author contribution statement

CA wrote the initial draft of the manuscript and produced the figure and tables. KEO edited and revised the manuscript, figure, and tables. Both authors approved the manuscript for submission.
